# The effect of the Yara smartphone application on anxiety, sleep quality, and suicidal thoughts in patients with major depressive disorder in Iran: a randomized controlled trial

**DOI:** 10.1186/s12888-024-05688-1

**Published:** 2024-03-27

**Authors:** Zeinab Soltani, Naser Parizad, Moloud Radfar, Vahid Alinejad, Mohammad Arzanlo, Mahmonir Haghighi

**Affiliations:** 1 Department of psychiatric nursing, Faculty of Nursing and Midwifery, Urmia University of Medical Sciences, Urmia, Iran; 2 Patient Safety Research Center, Clinical Research Institute, Faculty of Nursing and Midwifery, Urmia University of Medical Sciences, Urmia, Iran; 3 Department of Biostatistics, school of medicine, Urmia University of Medical Sciences, Urmia, Iran; 4 Department of psychiatry, school of medicine, Khoy University of Medical Sciences, Khoy, Iran; 5 Department of Psychiatry, School of Medicine, Razi hospital, Urmia University of Medical Sciences, Urmia, Iran

**Keywords:** Major depressive disorder, Anxiety, Sleep Quality, Suicide, Thought, Smartphone Apps, Iran

## Abstract

**Background:**

Depression is one of the most common mental disorders that leads to anxiety, sleep disturbances, and suicidal thoughts. Due to the high cost of treatment and the reluctance of many patients to seek medical help, major depressive disorder (MDD) is becoming more prevalent. Therefore, alternative methods like smartphone applications can help prevent and improve depression symptoms. The present study aimed to determine the effect of the newly developed Yara smartphone application on anxiety, sleep quality, and suicidal thoughts in patients with MDD.

**Methods:**

This randomized controlled trial with a pretest-posttest design was conducted on Iranian patients with MDD in 2022. Sixty-four patients were recruited using convenience sampling and randomly assigned to two control and intervention groups. The intervention was conducted using the Yara smartphone application for three months. Data were collected using the Spielberger State-Trait Anxiety Inventory (STAI), Pittsburgh Sleep Quality Index (PSQI), and Beck Scale for Suicidal Ideation (BSSI). Data were first entered into IBM SPSS Statistics for Windows, version 22 (IBM Corp., Armonk, N.Y., USA) and then analyzed using descriptive and analytical statistics.

**Results:**

There was no statistically significant difference in the mean score of anxiety and sleep quality between the intervention and control groups before the intervention (*p* ≥ .05). However, this difference in the mean score of anxiety and sleep quality was statistically significant in the two groups after the intervention (p < .05). The results showed no statistically significant difference in the mean score of suicidal thoughts between the two groups before and after the intervention (p ≥ .05). The use of the Yara smartphone application had a significant positive effect on anxiety and sleep quality in depressed patients (*p* < .001). At the same time, it had no significant effect on suicidal thoughts (*p* ≥ .05).

**Conclusion:**

Considering the positive effect of using the Yara smartphone application on reducing anxiety and improving sleep quality in depressed patients, this application can help alleviate the problems of depressed patients alongside existing treatment methods. Thus, this application is recommended for this group of patients in psychiatric clinics and departments. The Yara application's effectiveness was not approved on suicidal thoughts in this study so that further investigation would be necessary.

**Trial Registration:**

Iranian Registry of Clinical Trial approval code (IRCT# IRCT20131112015390N7).

## Background

Depression is one of the most common mental disorders worldwide [1]. More than 350 million people suffer from this disorder worldwide, and it is predicted to rank highest in the disease burden by 2030 [1–3]. The prevalence of depression is 42.59% of the general population in Iran [4]. Depression increasingly impacts the global disease burden [5], so people with severe depression report serious problems in all aspects of their lives, including occupational, home, and social activities [6]. Depression leads to social and psychological issues such as anxiety, fear of being in a group [7], social deprivation [8], increased mortality [9], feelings of worthlessness [10], suicide [1], sleep disorders, and high direct and social costs [11, 12]. Depression is one of the most expensive disorders to treat and increases the tendency towards physical illnesses and suicide, which causes youth mortality [13].

Pharmacotherapy has been approved as a standard treatment method for depression. However, fear of its side effects, such as dependence, causes patients to avoid medications [1]. On the other hand, non-pharmacological interventions are usually clinical or hospital-based and require weekly or monthly visits. Moreover, time constraints, transportation problems, and associated costs make it difficult for 70% of patients to attend regular psychotherapy sessions [14]. Meanwhile, depressed patients with physical problems are often socially isolated and stay home. Therefore, any action used at home for patients can lead to tremendous success rather than hospital-based methods [1, 15, 16]. Nowadays, smartphones and related applications offer a wide range of services that can benefit patients with mood disorders. One of these applications is mobile health applications that increase access to care, reduce stress, improve diagnosis, and expand the treatment of various psychiatric disorders [17]. These health applications reduce appointment waiting time and the need for face-to-face visits with a physician. The use of health applications is even more cost-effective than other methods [18]. However, only a few of these applications have focused on supporting individuals with mental health problems such as stress, anxiety, depression, post-traumatic stress disorder, and obsessive-compulsive disorder [19]. Given the widespread access to smartphones and their potential for self-management, it seems that the use of smartphones in patients with depression is becoming more important than ever before [20]. Regarding the prevalence and pandemic of COVID-19 as a highly contagious disease, remote nursing counseling and education through smartphone applications can be useful and reduce the patients' referral to hospitals, reducing the risk of exposure to this deadly disease [9]. In 2018, Deady et al. investigated the effect of the "HeadGear" smartphone application on treating depression symptoms for three months in Australia and found a positive effect on reducing the symptoms of patients with depression [6]. In another study in the United States in 2018, Pratap et al. investigated the effect of smartphone applications on depressed patients for six months and observed no positive impact on depression symptoms. They suggested further studies to comprehensively examine smartphone applications for patients with different cultures [21]. Therefore, there is no consensus about using smartphone applications to reduce depression symptoms, and further investigation is still needed. Given the high prevalence of depression in Iran [4, 22] and the world [23], the disabling and numerous symptoms of this disorder [4], and the easy access of depressed patients to smartphones, the research team designed a smartphone application (Yara) to investigate its impact on anxiety, sleep quality, and suicidal thoughts in patients with MDD. This study aimed to determine the effect of new developed YARA smartphone application on anxiety, sleep quality, and suicidal thoughts in patients with MDD. The research hypothesis was that using the Yara smartphone application would improve anxiety, sleep quality, and suicidal thoughts in patients with MDD.

## Methods

### Study design

This randomized controlled trial was conducted using a pretest-posttest design in 2021. The study was registered in the Iranian Registry of Clinical Trial (IRCT# IRCT20131112015390N7).

### Study setting and sampling

The participants included patients with MDD who were referred to Razi Hospital (in Urmia, West Azerbaijan) and the Psychiatry Clinic of Madani Hospital (in Khoy, West Azerbaijan) in Iran. Considering the data provided in a study by Collins et al. (2020) [24], the initial sample size was determined to be *n* = 32 for each group.

After obtaining approval from the Research Ethics Committee of Urmia University of Medical Sciences, the researcher visited the determined study settings and granted the introduction letter to their authorities. Sixty-four patients diagnosed with MDD in the past year who met the inclusion criteria were recruited to participate in the study using convenience sampling. The inclusion criteria consisted of the following: (a) willingness to participate in the study, (b) being able to use a smartphone, install the application, and work with it, (c) being diagnosed with MDD according to DSM-5 criteria, (d) being literate, and (e) not being hospitalized. The exclusion criteria included the following: (a) unwillingness to continue participation in the study and (b) being hospitalized during the study.

### Randomization

After obtaining the written informed consent and providing the necessary information about the study, the participants were randomly assigned to control and intervention groups using the simple randomization method. A pack of cards was used for random assignment, where patients who met the eligibility criteria selected either card A or B from the pack. Patients who selected card A were assigned to the control group, while those who picked card B were assigned to the intervention group.

### The procedure

The Yara application was installed on the smartphones of the patients in the intervention group, and the researcher provided necessary instructions on how to use it during a justification session in the hospital conference room. The process of using the application was thoroughly taught to the patients. The leading researcher evaluated the patients' understanding and use of the application. Each patient logged into the application in the researcher's presence and used its different parts. All questions and concerns of patients about how to use the application were answered. Compliance with the use of the application was monitored by the option of sending an email to the researcher. Every time the patient logged into the application; an email was automatically sent to the researcher. Necessary information about anxiety, sleep quality, suicidal thoughts, and how to complete the questionnaires was provided to both groups. The questionnaires were completed before and after the intervention in the researcher's presence. The intervention was conducted using the Yara application for three months (February – April 2022). The patients in the control group only received routine care for three months. Routine care received by patients of both groups included doctor visits, medication prescriptions, and psychiatric consultations once a month. The consultation usually takes about 30 minutes.

### Smartphone application (Yara)

**Fig. 1:**
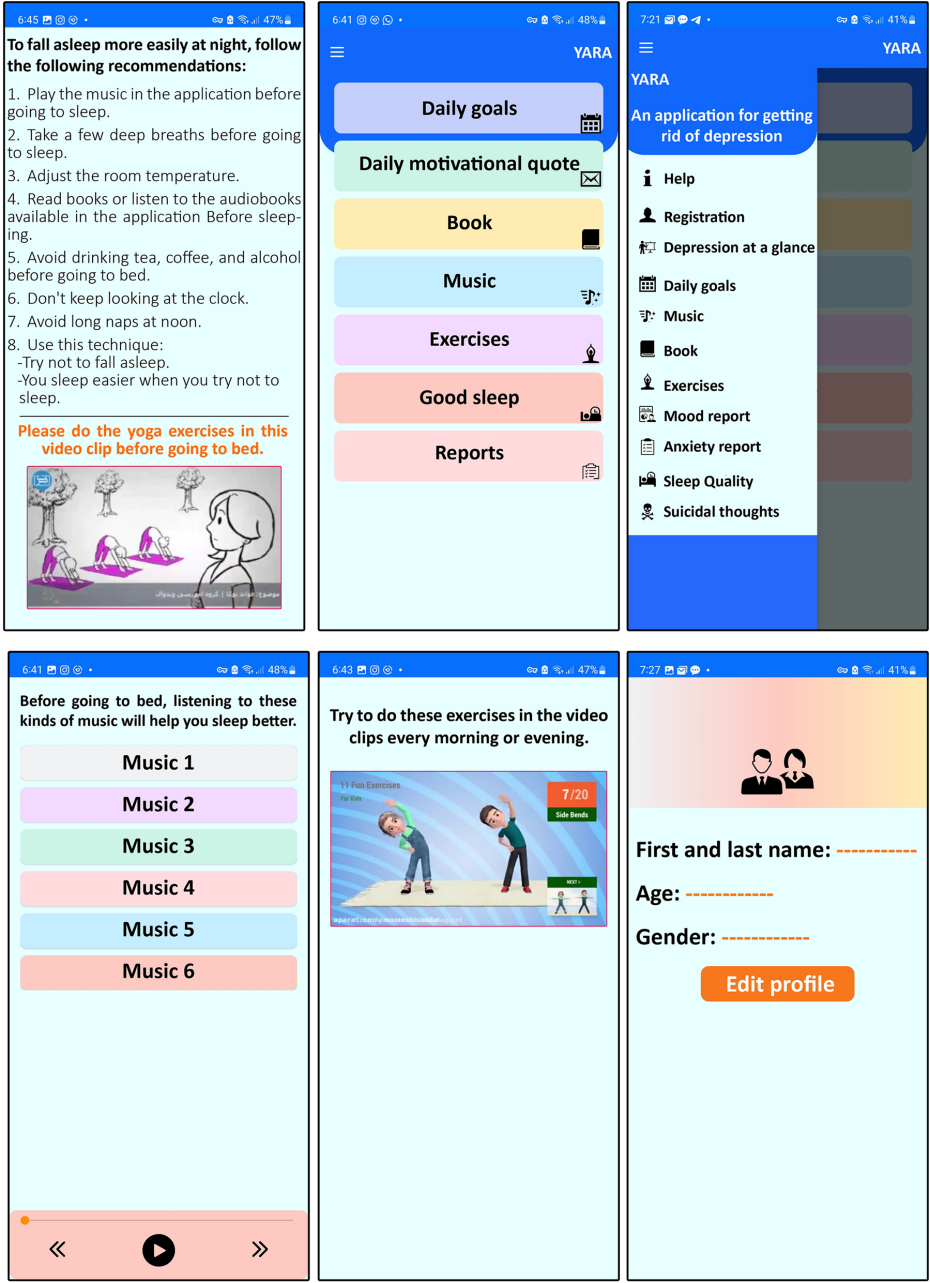
Images of the Yara smartphone application

Due to the popularity of smartphone applications in managing chronic diseases, the lack of consistent results in their application, and especially the emergence of COVID-19, the research team decided to design and use a new smartphone application for patients with depression. With the cooperation of a computer software programmer, the researcher designed the Yara application. At first, the researcher designed the application's content under the supervisor's supervision, using reliable psychology sites and reliable books and following the examples of good existing applications. The app's content includes a daily exercise educational video to increase the client's physical activity, morning meditation exercise, a yoga video for bedtime, and standard wordless soothing music from nature that helps the client to fall asleep more easily. It helps to increase the quality of sleep; several authentic personal development books available in PDF format can help change the client's attitude towards life, and a section that includes three tests for the daily report of patients' sleep quality, suicidal thoughts, and anxiety. In this application, the client receives motivational sentences daily and records her daily activities. The client reports daily anxiety, sleep quality, and suicidal thoughts in the application on the page related to each parameter and receives an automatic solution. Then, the software was designed and built attractively. Every time the client uses the application, an email is sent to the researcher to ensure that the client complies with the use of the application. The patient will be contacted if the patient does not use the application for two consecutive days. The researcher will access this data in the application after completing the intervention. (Fig. 1)

To check the validity and reliability of the Yara application, ten experts from different fields were used, including two psychiatrists, two psychiatric mental health nurses with Ph.D. degrees, two computer and software engineers, two psychologists, and two musicologists. Validation was done by the Delphi method. The designed smartphone application and the thesis proposal were installed on the professors' smartphones. After a week of evaluation, the lead researcher took their comments and suggestions and applied the requested changes. After the changes, it was again re-checked by the professors and approved. Then, the application was installed on five patients with depression, and it was confirmed in terms of face validity and comprehensibility. After ensuring the validity and correctness of the application, it was checked by the ethics committee, and after its approval, it was given to the intervention group.

### Data collection

In this study, data were collected using a demographic questionnaire (age, gender, marital status, education, and job status), the Spielberger State-Trait Anxiety Inventory (STAI), the Pittsburgh Sleep Quality Index (PSQI), and the Beck Scale for Suicidal Ideation (BSSI).

The STAI is used to measure anxiety levels. This inventory consists of two parts. The first assesses the individual's state anxiety (situational anxiety) and contains 20 questions on a 4-point Likert scale (Not at all = 1, somewhat = 2, moderately = 3, Very much so = 4). The second part assesses the individual's trait anxiety and consists of 20 questions on a 4-point Likert scale (Almost never = 1, Sometimes = 2, Often = 3, Almost always = 4). Finally, scores for both parts (state and trait) are separately calculated and recorded as a score between 20 and 80 for each individual. Patients' anxiety levels were categorized based on their scores, ranging from mild to severe anxiety. Spielberger et al. (1983) reported Cronbach's alpha coefficients for the state and the trait part of the inventory to be 0.92 and 0.90, respectively. The test-retest reliability coefficients for the state and the trait part were also calculated as 0.62 and 0.68, respectively [25]. In studies conducted to investigate the validity and reliability of this inventory in Iran, its reliability and validity coefficients were reported to be 0.91 and 0.90, respectively. Accordingly, the STAI was indicated to have high levels of validity and reliability in the Iranian population [26]. In this study, the scale's reliability was confirmed in a pilot study with Cronbach's alpha coefficient of 0.92.

The BSSI was developed by Aaron Beck in 1961. This scale consists of 19 items and is scored on a three-point Likert scale ranging from "Not at all true = 0" to "Definitely true = 2". The sub-scales include five questions about the desire to die, seven questions about readiness for suicide, and five questions about actual suicide intent. Questions 18 and 19 are not included in the scoring. The individual's total score ranges from 0 to 38 and is calculated by adding the scores. Scores of 0-5 indicate suicidal ideation; scores of 6-19 indicate suicidal readiness; and scores of 20-38 indicate suicidal intent. The BSSI has been shown to have correlation coefficients ranging from 0.90 to 0.94 with standardized tests for depression and suicidal tendencies. The BSSI has also been indicated to have a correlation coefficient ranging from 0.64 to 0.75 with the Beck Depression Inventory and the Beck Hopelessness Scale. The reliability of the BSSI was assessed using the internal consistency and the test-retest reliability method, based on which its Cronbach's alpha coefficient was obtained from 0.87 to 0.97, and its test-retest reliability coefficient was calculated to be 0.54 [27]. In Iran, the BSSI was validated in a study conducted on soldiers by Anisi et al. (2005) with a concurrent validity coefficient of 0.76 and Cronbach's alpha coefficient of 0.95 [28]. A pilot study confirmed the scale's reliability with Cronbach's alpha coefficient of 0.91 in this study.

The PSQI was first developed by Dr. Buysse at the Pittsburgh School of Medicine in 1989. This tool consists of 18 items, all scored on a 4-point Likert scale ranging from 0 to 3. The questionnaire has seven sub-scales, including subjective sleep quality, sleep latency, sleep duration, sleep efficiency, sleep disturbance, use of sleep medication, and daytime dysfunction. To score the PSQI, participants must first assign one of three scores to each of the 18 items. A total score of higher than 5 indicates poor sleep quality. The internal consistency of this tool was approved with a Cronbach's alpha coefficient of 0.83, and its validity was reported to be appropriate by the scale developers, with a sensitivity of 89.6% and specificity of 86.5% [29]. In this study, the tool's reliability was approved in a pilot study with a Cronbach's alpha coefficient of 0.89.

### Data analysis

**Fig. 2:**
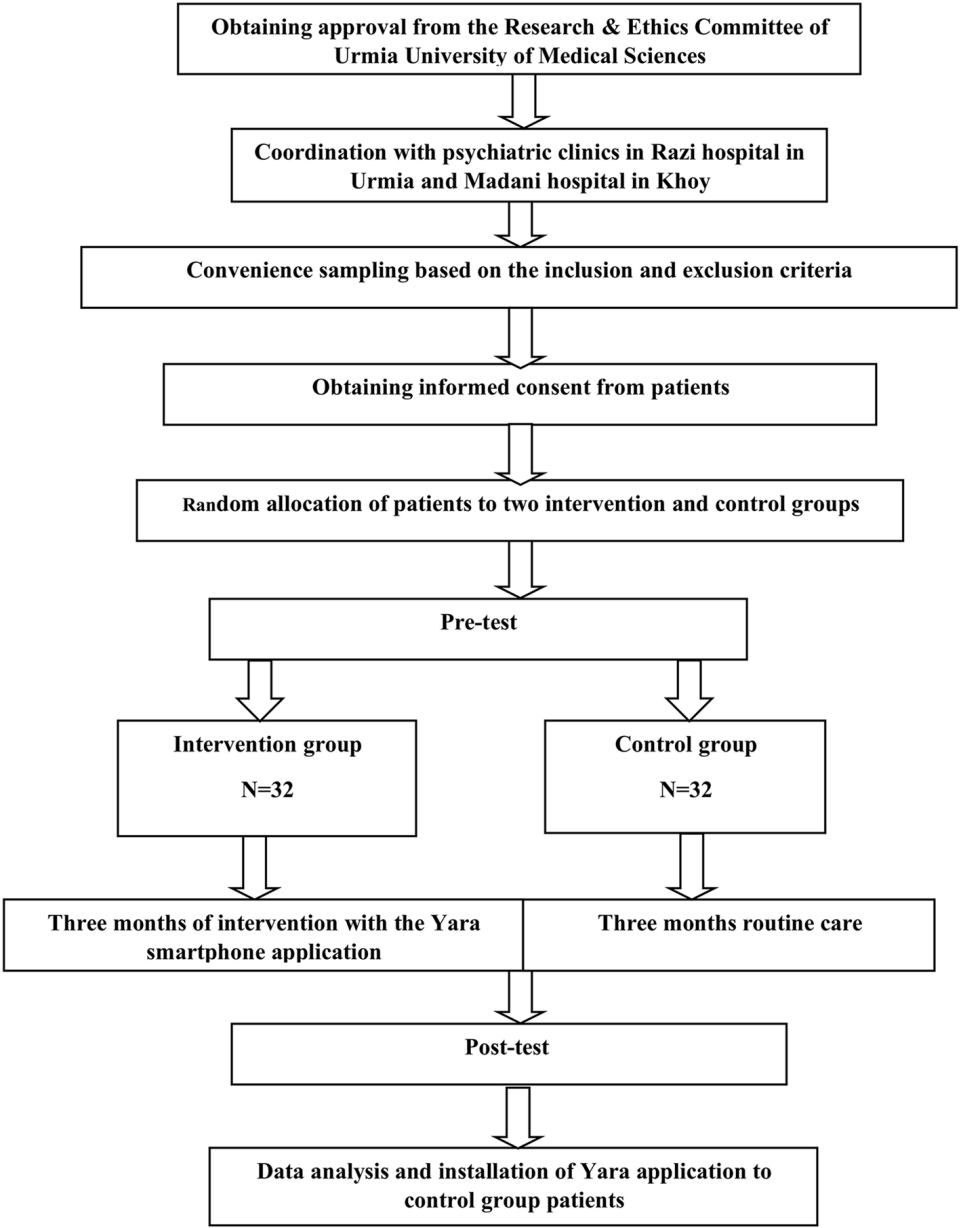
Flow chart of research implementation

Data were analyzed in IBM SPSS Statistics for Windows, version 22 (IBM Corp., Armonk, N.Y., USA). The frequency of characteristics was presented as number (%), and quantitative results as mean (±standard deviation), independent t, paired t, Mann-Whitney U, and Wilcoxon tests were performed to analyze data. All p values were two-tailed, and the significance level was considered as *p* < 0.05 (Fig. 2).

## Results

There was no sample attrition in this study, so 64 patients were included in the analysis. The data distribution was examined using the Kolmogorov-Smirnov test, and the data distribution was normal except for suicidal thoughts. The independent samples t-test results showed no significant difference in demographic variables between the two groups (Table 1).

**Table 1:** Comparison of the qualitative demographic characteristics of patients between the intervention and control groups

Variables		Intervention group		Control group		Chisquare test
		N	%	N	%	
Age	15-20 years	7	70	3	30	X^2^ = 3.133 * P* = 0.536
	21-30 years	11	45.8	13	54.2	
	31-40 years	11	45.8	13	54.2	
	41-50 years	3	60	3	40	
	51-60 years	0	0	1	100	
Gender	Male	11	73.3	4	26.7	X^2^ = 2.267 * P* = 0.390
	Female	21	42.9	28	57.1	
Marital status	Married	22	53.7	19	46.3	X^2^ 0.611 * P* = 0.434
	Single	10	43.5	13	56.5	
Education level	Some high school, no diploma	10	31.3	12	37.5	X^2^ = 2.610 * P* = 0.271
	High school diploma	17	53.1	11	34.4	
	College degree	5	15.6	9	28.1	
Job status	Employed	15	46.9	15	46.9	X^2^ =0.48 * P* = 0.599
	Unemployed	17	53.1	17	53.1	

There was no statistically significant difference in the mean scores of anxiety and its dimensions between the two groups before the intervention (*p* ≥ .05). However, this difference was shown to be statistically significant after the intervention (*p* < .001) (Table 2).

**Table 2:** Comparison of the mean score of anxiety and its dimensions between two intervention and control groups before and after the intervention

Variable	Time	Intervention group	Control group	Mean difference Control intervention	Independent *t* -test
		Mean±SD^c^	Mean±SD		
State anxiety	Pre-Interv. ^a^	59.38±4.29	59.69±2.98	- 0.313	*t* = - 0.339 df = 62 p = 0.736
	Post- Interv. ^b^	39.66±3.27	60.63±3.07	- 20.969	*t* = 26.466 df = 62 *p* < 0.001
Trait anxiety	Pre-Interv.	60.06±3.89	59.41±3.93	0.656	*t* = 0.671 df = 62 *p* = 0.504
	Post- Interv.	40.41±3.87	60.94±4.35	- 20.531	*t* = - 19.960 df = 62 *p* < 0.001
Total anxiety	Pre-Interv.	119.44±5.62	119.09±4.61	0.344	*t* = 0.268 df = 62 *p* = 0.790
	Post- Interv.	80.06±5.59	121.56±5.33	- 41.500	*t* = 30.375 df = 62 *p* < 0.001

^a^*Pre-interv* Pre-intervention

^b^*Post-interv* Post intervention

^c^*SD* Standard deviation

There was a statistically significant difference between the baseline and the post-intervention mean scores of anxiety in the intervention group (*p* < .05). However, this difference was not statistically significant in the control group (*p* = .084) (Table 3).

**Table 3:** Comparison of the mean score of anxiety in the intervention and control groups before and after the intervention

Anxiety	Pre-Interv.^a^	Post- Interv.^b^	The mean difference (pre-post intervention)	Paired t tests
	Mean±SD^b^	Mean±SD^b^		
Intervention group	119.44±5.62	80.06±5.59	39.38	*t* = 25.930 df = 31 *P* < 0.001
Control group	119.09±4.61	121.56±5.33	- 2.47	*t* = - 2.059 df = 31 *P* = 0.084

^a^*Pre-interv* Pre-intervention

^b^*Post-interv* Post intervention

^c^*SD* Standard deviation

There was no statistically significant difference in the mean score of sleep quality between the two groups before the intervention (*p* ≥ .05). However, this difference in the mean score of sleep quality was statistically significant after the intervention (*p* < .05). There was a statistically significant difference between the baseline and the post-intervention mean score of sleep quality in the intervention group (*p* < .05). At the same time, this difference was not statistically significant in the control group (*p* = .942) (Table 4).

**Table 4:** Comparison of the mean score of sleep quality between two intervention and control groups before and after the intervention

Variable	Time	Intervention group	Control group	Independent t-test
		Mean±SD^c^	Mean±SD	
Sleep quality	Pre-Interv.^a^	12.31±4.37	12.94±4.78	*t* = - 0.546 df = 62 *P* = 0.587
	Post- Interv.^b^	13.03±4.25	4.06±2.51	*t* = - 10.282 df = 62 *P* < 0.001
	Paired t-test	*t* = 8.585 df = 31 *P* < 0.001	*t* = - 0.073 df = 31 *P* = 0.942	

^a^*Pre-interv* Pre-intervention

^b^*Post-interv* Post intervention

^c^*SD* Standard deviation

According to the results of the Mann-Whitney *U* test, there was no statistically significant difference in the mean score of suicidal thoughts and its dimensions between the intervention and control groups before the intervention (*p* ≥ .05). Moreover, there was no significant statistically significant difference in the mean score of suicidal thoughts and its dimensions between the two groups after the intervention (*p* ≥ .05) (Table 5).

**Table 5:** Comparison of the mean score of suicidal thoughts and its dimensions between the two intervention and control groups before and after the intervention

Variables	Time	Intervention group	Control group	Mean difference (Intervention-control)	Mann-Whitney U test
		Mean±SD^c^	Mean±SD		
Desire to die	Pre-Interv. ^a^	0.41±1.34	0.38±1.48	0.03	Z = - 0.404 *p* = 0.686
	Post- Interv. ^b^	0.31±1.03	0.34±1.36	- 0.03	Z = - 0.375 *p* = 0.708
Readiness for suicide	Pre-Interv.	0.69±2.25	0.44±1.74	0.25	Z = - 0.462 *p* = 0.644
	Post- Interv.	0.56±1.80	0.41±1.64	0.15	Z = - 0.447 *p* = 0.655
Actual suicide intent	Pre-Interv.	0.53±1.72	0.19±0.780	0.34	Z = - 0.534 *p* = 0.593
	Post- Interv.	0.25±0.803	0.23±0.870	0.03	Z = - 0.404 *p* = 0.686
Suicidal thoughts	Pre-Interv.	1.63±5.148	1.00±3.935	0.63	Z = - 0.519 *p* = 0.603
	Post- Interv.	1.22±3.850	1.09±4.306	0.13	Z = - 0.375 *p* = 0.708

^a^Pre-interv Pre-intervention

^b^Post-interv Post intervention

^c^SD Standard deviation

According to the Wilcoxon signed-rank test, there was no statistically significant difference between the baseline and the post-intervention mean score of suicidal thoughts and its dimensions within the intervention group (*p* ≥ .05). Similarly, the same difference was not statistically significant within the control group (*p* ≥ .05) (Table 6).

**Table 6:** Comparison of the mean scores of suicidal thoughts in the intervention and control groups before and after the intervention

Suicidal thought	Pre-Interv.^a^	Post- Interv.^b^	The mean difference (pre-post intervention)	Wilcoxon tests
	Mean±SD^c^	Mean±SD^c^		
Intervention group	1.63±5.148	1.22±3.850	0.41	Z = - 1.604 P = 0.109
Control group	1.00±3.935	1.09±4.306	O.09	Z = - 1.342 P = 0.180

^a^*Pre-interv* Pre-intervention

^b^*Post-interv* Post intervention

^c^*SD* Standard deviation

## Discussion

The findings showed that the intervention and control groups had no statistically significant difference in demographic variables, including age, gender, marital status, education level, and Job-status that could affect the study results. Therefore, a significant difference between the baseline and post-intervention means anxiety scores in the intervention group were due to the Yara application's positive effect.

The results revealed that using the Yara application effectively reduced anxiety in patients with MDD. It should be noted that utilizing the Yara smartphone application was effective in reducing psychological problems such as anxiety because this application was developed based on the management of attention, relaxation, and self-control. Yoga and exercise videos are available in the application, which can help patients reduce stress. Moreover, using soothing instrumental music (such as sounds and melodies from nature) in this application can help reduce anxiety and promote restful sleep among patients [30]. When patients use the application, they become more aware of their surroundings and events, feel more in control, and can better control their thoughts, actions, and emotions. On the other hand, negative emotions can always disturb the mental state of patients and cause them to experience anxiety with constant thoughts about it [31]. This application can reduce anxiety using its emotion-control feature and the consideration of the current situation. In Iran, Borjalilu et al. (2016) also found that mobile phone applications can effectively reduce anxiety [32]. Among studies conducted in this area, Deady et al. (2020) reached similar results and found that smartphone applications can reduce depression symptoms and successive incidents of depression among Australian employees [33]. In the U.S.A., Longyear and Kushlev (2021) reviewed 18 clinical trials in a systematic review and reported that smartphone applications can reduce anxiety, depression, and stress [34].

The results indicated that using the Yara smartphone application had no significant effect on reducing suicidal thoughts in patients with MDD. O'Toole et al. (2019) also found that using a smartphone application did not significantly reduce depression and suicidal thoughts in patients [35]. Tighe et al. (2017) observed a significant decrease in depression symptoms after using the Ibobbly application but no significant reduction in suicidal thoughts or ideation [36]. Denneson et al. (2019) demonstrated that using smartphone applications alone is ineffective in alleviating depression symptoms and suicidal thoughts, but they can reduce depression symptoms and suicidal ideation severity if mediated through coping self-efficacy [37]. One of the most significant challenges to suicide prevention is that many known risk factors that predict changes in suicidal ideation over months/years (e.g., hopelessness) cannot be prevented over hours/days. This highlights a gap in our ability to prevent suicide in the short term [38]. Another plausible reason for the lack of effect of the smartphone application on suicidal thoughts can be the non-compliance of these patients with the interventions related to suicide prevention in the application. In the meta-analysis study conducted in Australia, Witt et al. (2017) reported that compliance with interventions related to suicidal thoughts was low in most studies [39]. Adherence to interventions related to suicidal thoughts was also poor during the long-term follow-up period. Over half (64%) of the participants did not access the intervention during the one-month follow-up period in the previous study[40]. This suggests that using digital innovations, including smartphone applications, may not be sufficient to sustain these interventions in the long term. It seems that the Yara application did not successfully improve suicidal thoughts in three months, so it is recommended to consider a longer time in employing suicide prevention applications in the future. Additionally, some studies have discussed the positive role of some applications in causing depression symptoms. Yang et al. (2010) reported that mobile applications and games can induce risky behaviors [41]. Khasawneh et al. (2020) also found that mobile phones, applications, and social media can induce suicidal thoughts [42]. Despite the above, numerous studies have approved the positive effect of smartphone applications in this regard [43, 44]. In a systematic review, Malakouti et al. (2020) mentioned the conflicting results regarding the effect of smartphone applications on suicidal thoughts and recommended conducting further studies in this regard [45].

The results indicated that the use of mobile phone applications had a positive effect on the sleep quality of patients and improved their sleep. The Yara application increased patients' awareness and knowledge by providing scientific content on sleep and the consequences of sleep disorders. It also helped them improve the quality of their sleep by offering physical exercise, soothing music tracks, and yoga. In line with our study results, in the U.S.A., Choi et al. (2018)found that some applications can effectively improve sleep quality due to their predetermined quality criteria, content, and performance for sleep management [46]. In a study conducted in Germany, Fietze (2016) also concluded that mobile applications can help individuals increase their awareness of sleep health and identify people with poor sleep quality compared to those who suffer more from paradoxical insomnia [47]. Karsaneh et al. (2022) conducted a review study in Jordan and concluded that existing applications for self-management of sleep disorders or improving sleep quality may be valuable, but they must be developed in quality and content [48]. Many people rely on sleeping pills as the most suitable solution for getting rid of sleep disorders. However, long-term use of such drugs has led to other problems and resulted in chronic insomnia. Clinical psychologists use various forms of psychotherapy to help individuals improve and maintain their sleep patterns [49]. It should be noted that many individuals choose natural treatments for depression and avoid pharmacotherapy. Coping with depression naturally can be effective if proper nutrition and better sleep are accompanied. The Yara application combines various techniques such as yoga, motivational speeches, and soothing music tracks to help users overcome depression naturally.

One of the limitations of this study was the simultaneous occurrence of the COVID-19 pandemic and patients' fear of catching it during the briefing session. This problem was addressed by providing personal protective equipment such as masks, disposable gloves, disinfectants, and face shields. Another limitation of the study was the possibility of using other measures to reduce the symptoms of depression by patients that were out of the researcher's hands. Another study limitation was the lack of long-term patient follow-up after the intervention period. This application is specially made for Iranian culture and the Persian language, so it may not apply to the general population. Therefore, the necessary changes for its internationalization and validation should be done. This study was conducted in a limited environment with a small sample size, which may pose challenges in generalizing the results. Therefore, it is recommended to conduct studies with larger sample sizes in diverse environments to examine the effects of intervention better. Additionally, it is suggested to investigate the effect of the Yara smartphone application on other symptoms of depression.

## Conclusion

The Yara application is a suitable solution for improving anxiety and sleep quality in patients with MDD. Digital technologies allow smartphones to be transformed into devices that can provide cost-effective mental health services and reduce users' stress. Using smartphone applications was ineffective in reducing suicidal ideation in this study. Many known risk factors that predict suicidal thoughts over months/years (e.g., hopelessness) cannot be prevented over hours/days. It is recommended to consider a longer time in employing suicide prevention applications in the future.

## Data Availability

The datasets generated during and/or analyzed during the current study are available from the corresponding author on reasonable request.

## Abbreviations

MDD: Major depressive disorder
STAI: Spielberger State-Trait Anxiety Inventory
PSQI: Pittsburgh Sleep Quality Index
BSSI: Beck Scale for Suicidal Ideation
SPSS: Statistical Package for the Social Sciences
COVID-19: coronavirus disease 2019
